# Mechanical Enhancement of Polychloroprene Adhesives via Reinforcement with Aluminum Oxide Nanofibers

**DOI:** 10.3390/polym17223064

**Published:** 2025-11-19

**Authors:** Il’ya Bril’, Anton Voronin, Yuri Fadeev, Ayraana Kuular, Marat Nureev, Fedor Ivanchenko, Mikhail Sumunin, Egor Moskvichev, Ivan Nemtsev, Sergey Dorbosmyslov, Alexandr Samoilo, Stanislav Khartov

**Affiliations:** 1 Federal Research Center “Krasnoyarsk Science Center”of the Siberian Branch of the Russian Academy of Sciences, 660036 Krasnoyarsk, Russia; a.voronin1988@mail.ru (A.V.); daf.hf@list.ru (Y.F.); ayraana.kuular@mail.ru (A.K.); orion-leo@mail.ru (F.I.); michanel@mail.ru (M.S.); ivan_nemtsev@mail.ru (I.N.); dobrosmislov.s.s@gmail.com (S.D.); stas_f1@list.ru (S.K.); 2 Regional Educational and Scientific Center “Security”, Bauman Moscow State Technical University, 105005 Moscow, Russia; 3 School of Engineering and Construction, Siberian Federal University, 660036 Krasnoyarsk, Russia; asamoylo@sfu-kras.ru; 4 LLC Research and Production Company «Spectehnauka», 660043 Krasnoyarsk, Russia; 5 OOO “NANOSINTEZ”, 660021 Krasnoyarsk, Russia; mapik86@mail.ru; 6 Federal Research Center for Information and Computational Technologies, 660049 Krasnoyarsk, Russia; jugr@ict.nsc.ru; 7 Kirensky Institute of Physics, Siberian Branch of the Russian Academy of Sciences, 660036 Krasnoyarsk, Russia

**Keywords:** chloroprene, alumina, composites, mechanical properties

## Abstract

In this study, we demonstrated chloroprene rubber (CR)-based composites with the addition of synthesized alumina nanofibers (AONF) with a high aspect ratio (>1000). AONF were characterized using transmission electron microscopy (TEM), scanning electron microscopy (SEM), and X-ray diffraction (XRD). AONF were introduced by pre-dispersion. The resulting chloroprene rubber/aluminum oxide nanofiber (CR/AONF) composites were subjected to tensile and shear adhesive bonding tests. The tensile test results for the CR/AONF composites are 81% greater than those of the original CR composite (0.85 MPa and 1.54 MPa, respectively). Shear adhesive bonding tests were conducted for glass and steel. CR/AONF demonstrates a 213% (for steel) and 262% (for glass) increase in shear strength. The main strengthening mechanisms are reinforcement, CR adsorption on the AONF surface, and crack arrest. These results may expand our understanding of the potential of sealant strengthening using AONF.

## 1. Introduction

Chloroprene rubbers (CR), a class of polymers based on 2-chloro-1,3-butadiene, have been critically important specialty elastomers for several decades. Their usability stems from a unique, balanced combination of performance characteristics, which includes high mechanical strength and elasticity; resistance to oils, ozone, oxygen, and environmental conditions, and elevated temperatures [1,2]. CR have found wide application as structural materials in key industries due to this combination of properties. CR are used to produce conveyor belts for mining equipment, vibration- and sound-insulating elements, cable protective sheaths, diaphragms, seals, and cuffs in mechanical engineering, as well as high-adhesion adhesives and sealants [3].

Despite these advantages, the continuous tightening of technical requirements for the properties of polymeric materials highlights the need for targeted modification of existing compositions to overcome the inherent limitations of elastomers, particularly in the context of operation under extreme conditions, such as increased mechanical loads, intense abrasive wear, high temperatures, and aggressive chemical environments [4,5,6].

Traditional technologies for modifying the properties of CR with micro- and submicrofillers have systemic drawbacks. For example, filling with carbon black is the most common and effective strengthening method. Different grades of carbon black allow for a wide range of properties. Highly active carbon black (with small particle diameters and large surface areas) provides significant increases in strength, rigidity, and wear resistance due to the formation of a dense filler network and strong adhesive interactions at the phase interface [7,8]. However, this method has serious drawbacks: a sharp increase in the viscosity and elastic modulus of unvulcanized mixtures, which impairs their processability (processing during extrusion and calendering), leads to increased heat generation, and increases energy consumption. In addition, high soot contents (over 40–50 mass parts) often lead to loss of elasticity, increased hysteresis losses, and, as a consequence, strong heating of products under dynamic loads [9,10].

Filling with silicates (e.g., silicon dioxide) is also widely used, particularly when enhanced adhesion to metals and textiles is required. Silica fillers, especially those modified with silanes, are capable of creating an extensive network of hydrogen bonds, effectively reinforcing the polymer [11,12]. The main problems here are a sharp increase in vulcanization time and high hygroscopicity, requiring special storage conditions for the mixtures. As with carbon black, achieving significant reinforcement requires high degrees of filler, with all the attendant negative consequences for processability and elasticity.

Combination with other polymers (PVC, polyolefins, other rubbers), although it allows one to level out the disadvantages of one component due to the advantages of another, is associated with the risk of phase separation of the composition, instability of properties, and the complexity of managing the technological process of its production.

Thus, traditional methods of CR modification, while aiming to improve some properties, negatively affect others.

The nanomodification strategy opens up new, revolutionary possibilities for improving elastomeric materials. The integration of nanoscale fillers, which have orders of magnitude greater specific surface areas than their micron-sized counterparts, enables fundamental changes in the structure and properties of composites even at low (1–5 wt.%) filling levels [13]. The high surface energy of nanoparticles facilitates their intense interaction with polymer macromolecules, leading to the formation of an interfacial layer with molecular dynamics distinct from those of the polymer bulk. This leads to the formation of a spatial network that effectively redistributes mechanical loads and acts as a reinforcing framework, ultimately improving the strength, barrier, and thermal properties of the material [14,15].

A wide range of nanofillers for modifying polymer matrices is currently being actively studied, with each having specific advantages and limitations.

Multiwalled carbon nanotubes (MWCNTs) exhibit exceptional strength properties (tensile strength up to 100 GPa) and electrical conductivity [16]. Their high flexibility and aspect ratio (length/diameter > 1000) facilitate the formation of a three-dimensional reinforcing network in the polymer matrix. However, MWCNTs show a tendency to aggregation, they are expensive, and are not suitable for use in dielectric composites [17].

Graphene and its derivatives have a unique two-dimensional structure. They exhibit record-breaking values of specific surface area (~2630 m^2^/g), thermal conductivity (~5000 W/m K), and mechanical strength [18]. However, they are prone to aggregation, like MWCNTs. The anisotropy of the properties of graphene-containing composites can lead to non-uniform mechanical properties [19].

Aerosil (nanosized silicon dioxide) is one of the most widely used nanofillers due to its large specific surface area (150–400 m^2^/g) and relative availability [20]. Its spherical particles effectively increase hardness, elastic modulus, and wear resistance. However, its introduction sharply increases the viscosity of composites and their tendency to be hygroscopic. The isotropic morphology of the particles limits the maximum achievable level of reinforcement [21].

Nanosized calcium carbonate is attractive due to its low cost and ease of production [22]. Its particles can improve the mechanical properties and thermal stability of composites. However, its low aspect ratio and limited adhesion to polymer matrices require the use of surface modifiers [23].

Montmorillonite and other layered aluminosilicates can significantly improve the barrier properties and fire resistance of composites [24]. Key issues remain the difficulty of complete exfoliation of aggregates and the need to use compatibilizers to improve adhesion to non-polar matrices [25].

Aluminum oxide (Al_2_O_3_) nanofibers represent a highly promising filler, advantageously combining the advantages of various classes of nanomaterials while devoid of many of their drawbacks. Fibrous morphology with an aspect ratio > 1000 ensures the formation of a strong reinforcing framework at low concentrations (0.5–3 wt.%), leading to a significant increase in mechanical properties without a catastrophic drop in elongation at break [26].

The exceptional hardness of Al_2_O_3_ particles (9 on the Mohs scale) combined with the fibrous form results in a synergistic improvement in wear and abrasion resistance [27]. Unlike carbon nanomaterials, aluminum oxide is a dielectric and chemically inert, which ensures good compatibility with the polar groups of chloroprene rubber [28].

Thus, alumina nanofibers appear to be an optimal choice for modifying CR, allowing for significant strengthening, increased thermal stability, and wear resistance without compromising the processability of the processing [29].

The relevance of this study stems from the need to develop new, highly effective composite materials based on chloroprene rubber with an expanded range of performance characteristics due to the synergistic effect of introducing alumina nanofibers.

In this work, we synthesized alumina nanofibers using a melt method and investigated the structure and properties of chloroprene rubber-based nanocomposites modified with alumina nanofibers. The composites demonstrated significant improvements in mechanical properties (tensile strength and shear strength of adhesive joints). These results expand the potential for strengthening adhesives and sealants using AONF.

## 2. Materials and Methods

### 2.1. Alumina Nanofiber (AONF) Synthesis

AONF were obtained from ENAW-1199 aluminum using molten aluminum oxidation technology. At the first stage, metallic aluminum is melted and various additives are injected into the melt. During the second stage, AONF were synthesized in the presence of O_2_ from the resulting melt [30]. The obtained nanofibers were synthesized with a highly oriented texture and had an extremely high aspect ratio (>1000) of length, in the range of centimeters at nanometer diameters. In the work, nanofibers were used in the form of a suspension; for this purpose, they were treated with ultrasound in ethyl acetate for 5 min [31,32,33].

### 2.2. AONF Characterization

#### 2.2.1. Microscopy

The morphology and geometric characteristics of individual AONF were studied using transmission electron microscopy (TEM) with a Hitachi HT 7700 (Hitachi, Tokyo, Japan) operating at an accelerating voltage of 40–300 kV. A suspension of ANF was diluted 100 times with ethanol, and then a carbon-coated copper mesh was immersed to transfer a small amount of ANF.

#### 2.2.2. XRD

The powder diffraction data of samples for Rietveld analysis were collected at room temperature with a Haoyuan DX-2700BH (Haoyuan, Dandong, China) powder diffractometer (analytical equipment of Krasnoyarsk Regional Center of Research Equipment of Federal Research Center “Krasnoyarsk Science Center SB RAS”) with Cu-Kα radiation and a linear detector. The step size of 2θ was 0.01°, and the counting time was 0.2 s per step. Therefore, these structures were taken as the starting model for Rietveld refinement, which was performed using TOPAS 4.

### 2.3. AONF Characterization

#### 2.3.1. CR Composition Synthesis

When studying the reinforcing properties of aluminum oxide nanofiber for an adhesive joint made of chloroprene rubber, the following composition was chosen as a basis [34]. The initial components were in the following ratio: 6.5% by weight high-crystallization-rate polychloroprene, 6.8% by weight medium-crystallization-rate polychloroprene, 1.0% by weight magnesium oxide, 1.6% by weight zinc oxide, 5.5% by weight butylphenol-formaldehyde resin, 0.25% by weight agidol, 0.08% by weight water, and 78.27% by weight ethyl acetate. The adhesive composition was manufactured step by step according to the following procedure:

Step 1. Dissolving polychloroprene.

Solvent (50% of the total volume) was loaded into a reactor with a stirrer. Polychloroprene was added in portions (20% by weight) at 25–30 °C and stirred for 2–3 h until completely dissolved (stirrer speed was ~200 rpm).

Step 2. Adding oxides and resin.

Next, magnesium and zinc oxides, pre-sifted through a 100 μm sieve, were added with constant stirring for 30 min. Then, butylphenol-formaldehyde resin and agidol were added. The mixture was heated to 40–50 °C to accelerate dissolution (1 h).

Step 3. Homogenization and addition of water.

The remaining solvent was added and stirred for 1 h. Water (0.08%) was added to stabilize the viscosity. The resulting mixture was filtered through a 50 μm mesh.

#### 2.3.2. Microscopy

The morphology of CR and CR/AONF composites was studied by scanning electron microscopy (SEM) on a SU3500 microscope (Hitachi, Tokyo, Japan) at an accelerating voltage of 20 kV. EDX images and spectra were obtained on a SU3500 microscope equipped with an energy-dispersive X-ray spectrometer XFlash 430 (Brucker, Billerica, MA, USA).

### 2.4. Thermomechanical Test

#### 2.4.1. Shear Strength Tests

For shear testing, the bonding surface (steel and glass) in the form of 60 × 20 × 2 mm plates was treated with ethyl alcohol and dried for 20 min. A thin layer of adhesive was then applied to the plates, covering an area of 20 × 30 mm, and maintained under normal conditions for 10 min. The plates were then glued together and placed under a pressure of 13 kPa. They were maintained under load for 24 h. After removing the load, the specimens were dried under normal conditions for 21 days. The specimens were tested according to [35].

Shear strength tests were performed on an Instron 3369 (Instron, Norfolk, MA, USA) according to [35]. The crosshead speed was 0.2 mm/min. We tested five samples for each concentration. We averaged the numerical results and plotted the graph for each concentration.

#### 2.4.2. Tensile Tests

For tensile testing, the CR composite was poured into disposable polystyrene molds (filled to a depth of 1 mm). The molds were dried under normal conditions for 21 days. The molds were then disassembled, and specimens were cut out for testing.

Tensile testing was performed on a REM 1-U-A universal testing machine (METROTEST, Moscow, Russia). Specimens were tested according to [36]. The crosshead speed was 0.02 mm/min. Deformations were calculated using an extensometer. We tested five samples for each concentration. We averaged the numerical results and plotted the graph for each concentration.

#### 2.4.3. TGA, DTGA, DSC Analysis

The DSC, TGA, and DTGA data were obtained using SDT Q600 (TA instruments, New Castle, DE, USA). Samples were placed in a platinum crucible, mounted on the sensor along with a reference crucible, and heated at 20 °C/min in an ambient air flow of 50 mL/min to a temperature of 800 °C. Heating rate: 20 °C/min. Blow-off: 50 mL/min. Atmosphere: air.

#### 2.4.4. DMA Analysis

Thermomechanical study of the viscoelastic properties of the samples was conducted using a dynamic mechanical analyzer Q800 (TA Instruments, New Castle, DE, USA). Measurement of storage modulus (E’) of a sample was carried out using a compression clamp and a linear temperature scanning in the heating mode from 25 to 150 °C at a 3 °C/min rate. The frequency of dynamic loading of the sample was 1 Hz, and the relative deformation was less than 0.1%. The samples were investigated in the form of plates with a size of 30 mm × 12.5 mm × 0.65 mm.

## 3. Results and Discussion

### 3.1. Structure of Aluminum Oxide Nanofibers

**Figure 1:**
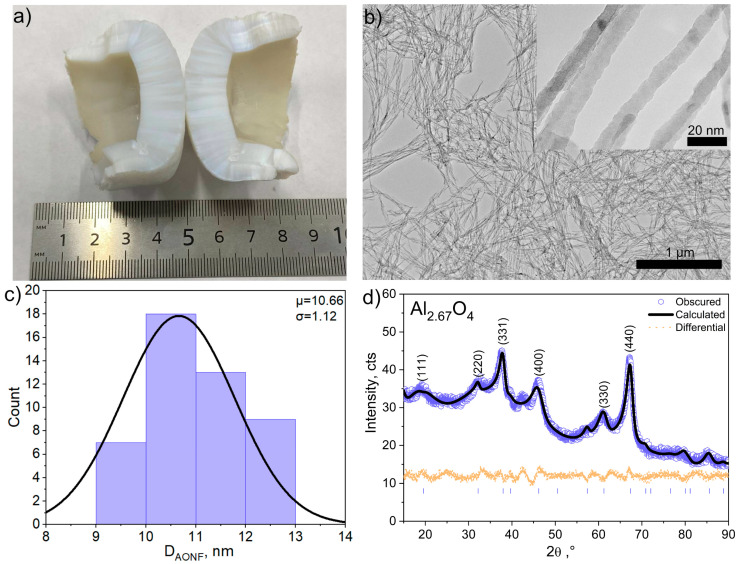
Photo of AONF (**a**), TEM images of AONF (**b**), histogram of AONF diameter distribution (**c**), XRD spectra of AONF (**d**).

The AONF we synthesized are blocks of aluminum oxide nanofibers. Figure 1a shows a photograph of a block of the nanofibers we synthesized. The morphology of the AONF was studied using a transmission electron microscope (TEM). The microscopy results are shown in Figure 1b. The image shows that the fiber diameters do not exceed 20 nm. The fiber length is several times greater than the diameter.

To further study their steric parameters, we constructed a histogram of the AONF diameter distribution. The diameters are normally distributed. The average diameter is 10.66 ± 1.12 nm. The AONF we synthesized have a smaller diameter than nanofibers synthesized by electrospinning [37,38].

The results of X-ray diffraction analysis of aluminum oxide (Al_2_O_3_) nanofibers are shown in Figure 1c. The analysis of the diffraction pattern revealed the presence of characteristic reflections corresponding to the γ-phase of Al_2_O_3_ (JCPDS No. 77-0396), namely (111), (220), (311), (400), (333), and (440), which are observed at 2θ angles of ~18.83°, 31.85°, 37.48°, 46.04°, 60.71°, and 67.07°, respectively [39]. It was established that the studied material crystallizes in a cubic syngony (Fd3m) with the unit cell parameter a = b = c = 7.906 Å, which is in good agreement with the literature data for γ-Al_2_O_3_.

The method of introducing AONF into the chloroprene rubber matrix affects its distribution within the polymer. Typically, nanomaterials are introduced into the polymer via pre-dispersion. Dispersion allows for uniform distribution of the nanomaterial within the polymer, preventing the formation of agglomerates that could negatively impact the mechanical properties of the materials. For example, Fan [40] used water as a dispersant for MWCNT. There are no data in the literature on methods for introducing AONF into CR.

**Figure 2:**
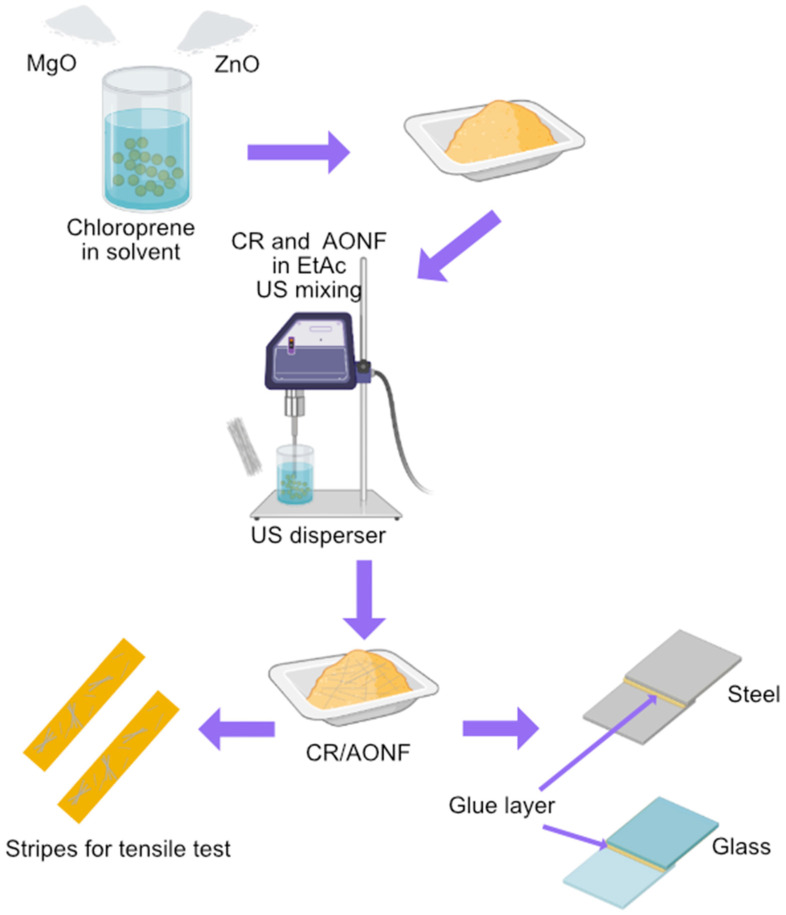
Sample preparation scheme.

We used pre-dispersion to introduce AONF into the CR composite. The flow chart for introducing AONF is shown in Figure 2.

We chose this method of introduction because it allows us to achieve the division of AONF into blocks of 3–8 fibers, which allows us to reduce the average particle size of the filler.

### 3.2. Microscopy Results

**Figure 3:**
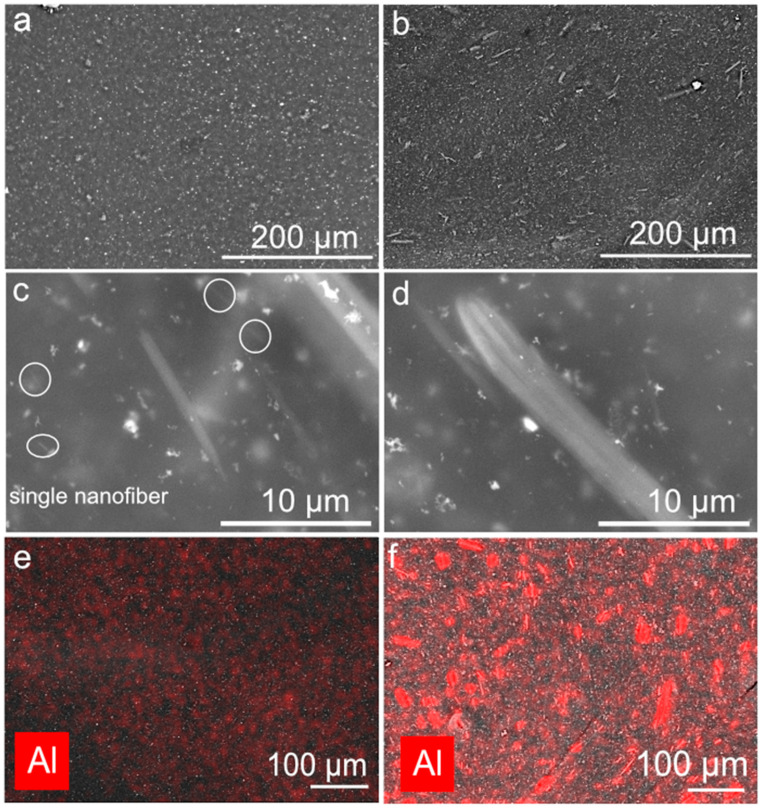
SEM image of pure CR composite (**a**) and CR/AONF (1 wt.%) (**b**–**d**); EDX images of CR composite (**e**) and CR/AONF (**f**).

To characterize the dispersion quality, we conducted an SEM study of the samples. Figure 3 shows the SEM and EDX images.

As we can see, the CR composite initially contains no aluminum (Figure 3a). The contrasting dots represent the mineral content of the CR composite in the form of ZnO and MgO. A different picture is observed in the CR/AONF composite. Figure 3b shows an SEM image of the bulk of the CR/AONF composite. We see that the AONF in the CR matrix are presented as a hierarchical structure containing both agglomerates and single AONF. For a more detailed examination of the AONF agglomerate sizes, we present the images in Figure 3c,d. The bulk of the CR/AONF composite contains both single AONF (highlighted by the white circle) and agglomerates of varying sizes. The average agglomerate size is approximately 300 nm.

The presence of two AONF fractions in a CR composite can affect both the thermal and mechanical properties of the CR/AONF composite.

## 4. Mechanical Properties of CR/AONF

The addition of AONF to the CR composite introduces a new dimension to this heterogeneous composition. In addition to influencing the kinetics and thermal deformation intensity, nanofillers significantly influence the mechanical properties of the composite. Beyond their direct influence through their characteristics, AONF creates another phase boundary. Unique phenomena occurring at this interface contribute to the behavior of the AONF/CR composite under load.

To evaluate the effect of AONF on mechanical properties, we conducted mechanical tests. We tensile tested adhesive strips and shear tested the bonded surfaces. We used glass and steel as the bonding surfaces.

**Figure 4:**
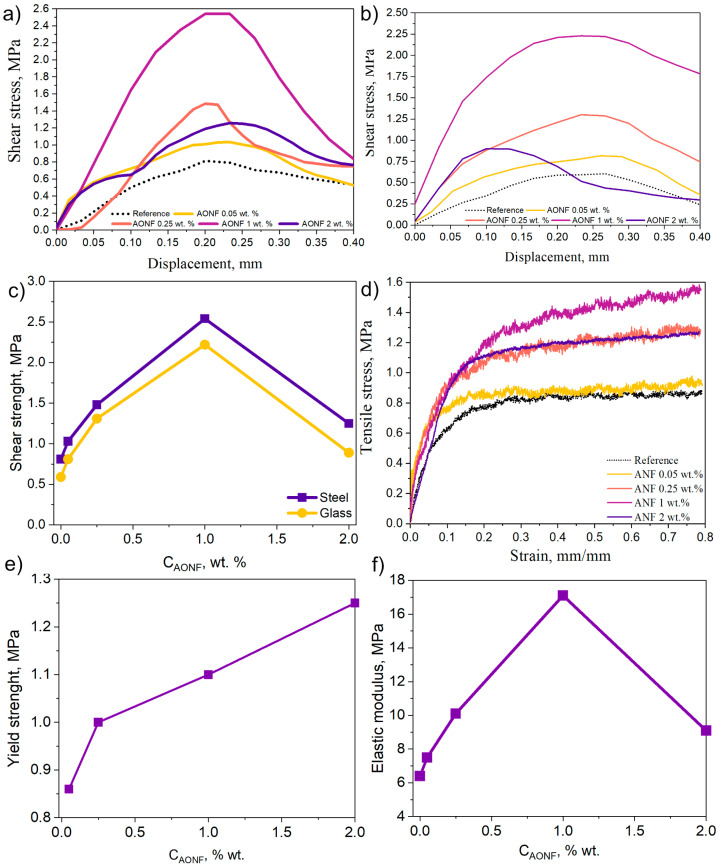
Results of shear tests of bonded steel plates (**a**), results of shear tests of bonded glass (**b**), shear strength (**c**), tensile stress (**d**), calculated yield strength (**e**), calculated elastic modulus (**f**).

Shear tests allow us to evaluate the applicability of the resulting AONF/CR composite in real-world applications. The shear test results for bonded steel plates are shown in Figure 4a, and the shear test results for bonded glass are shown in Figure 4b. The graphs show an increase in the maximum shear load with increasing concentration. For example, in the case of bonded steel plates, the peak shear force for the reference sample is 0.81 MPa, while for the AONF/CR sample with 0.05 wt.% AONF, it is 1.02 MPa (an increase of 25%). And in the case of bonded glasses, the shear force for the reference sample is 0.60 MPa, while for the sample with 0.05 wt.% AONF, it is 0.81 MPa (an increase of 35%). A further increase in concentration also leads to an increase in the peak shear load values for both bonded steel plates and glasses. Peak shear force values are demonstrated by AONF/CR composites with 1 wt.% AONF. For bonded steel plates, the shear stress is 2.54 MPa (an increase of 213%), while for bonded glass plates, it is 2.22 MPa (an increase of 263%). This is the optimal concentration for both cases. Strengthening of AONF can occur through the following mechanisms.

During deformation of the AONF/CR composite at the macroscopic level, deformation of the polymer matrix leads to the generation of shear stress on the AONF surface. Since the fibers are strongly bonded to the matrix (due to good adhesion), stress is transferred from the soft matrix to the stiff fibers. Thus, the fibers act as an internal reinforcement cage, preventing failure and deformation [32]. With the addition of 1 wt.% AONF, we observe a steep initial portion of the shear stress curve and a shift in the peak strength to the region of higher stresses for the modified samples.

The second strengthening mechanism involves the adsorption of CR on the large specific surface area of AONF [41]. This shell structure around each AONF behaves like a material with properties distinct from those of the CR matrix. The boundary phase often exhibits increased density and strength. During deformation, more energy is required to fracture it. The increase in elastic modulus and peak shear loads confirms the existence of this mechanism.

Furthermore, AONF dispersed throughout the matrix acts as a physical barrier to microcrack propagation. When a growing crack encounters AONF, it cannot pass directly through it (due to its high strength). It must either bend around it (which requires additional energy and increases the crack path) or pull the fiber out of the CR matrix (which also requires energy to overcome s forces). This process is repeated multiple times, significantly slowing failure. This is indicated by a flatter and more extended region after the peak strength (sample with 0.05% and 0.25 wt.% for steel plates and glass).

Further increasing the AONF concentration in the AONF/CR composite to 2 wt.% leads to a decrease in shear strength for both glass (0.91 MPa) and steel plates (1.25 MPa) relative to an AONF concentration of 1 wt.%. The negative effect of AONF at a concentration of 2 wt.% can be explained by the following mechanisms. A high AONF content physically “displaces” the volume of the CR matrix, which is the binding element responsible for plasticity and load redistribution. This makes the composite more brittle. Nanofibers, located too close to each other, prevent the matrix from deforming and absorbing energy. Instead, crack bridges form between them. This is evident in the shear plots for AONF/CR with 2 wt.%. The slopes of the plots after the peak become sharper than those for AONF/CR composites with a lower AONF content and the reference sample.

A second possible mechanism is weak adhesion at the interface. At high filler concentrations, it becomes impossible to ensure perfect adhesion over the entire surface of each fiber. Regions with weakened adhesive interactions form. Instead of stress transfer, AONF delaminates from the CR matrix. The resulting microvoids coalesce and lead to brittle fracture.

The third possible mechanism is related to the influence of the degree of AONF dispersion at the injection stage. The AONF fraction consists of directional bundles of 3–8 fibers. When the concentration reaches 2% by weight, the volume of AONF bundles in the AONF/CR composite reaches a level that prevents the CR polymer chains from moving freely. However, the applied force is sufficient to shear the AONF relative to each other within the bundles. This movement creates defects in the structure of the AONF/CR composite, which negatively impacts strength and leads to a decrease in the maximum shear force.

**Figure 5:**
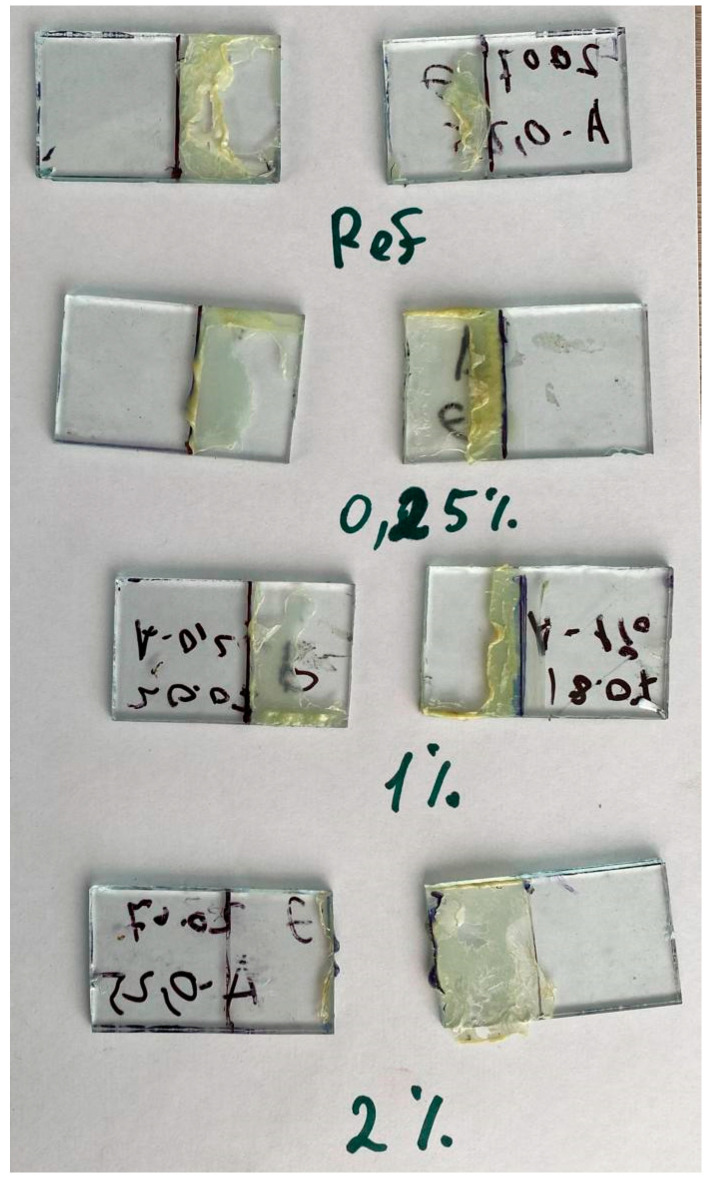
Photo of samples for shear strength test after tests.

In addition to influencing the strength properties of the CR matrix, AONF influences its adhesive interaction with the glass. As we previously noted, the relative strength increase for bonded glass is higher than for bonded steel plates (for samples containing 1% by weight AONF, the increase relative to the reference value was 213% for bonded steel plates and 263% for bonded glass). Taking this into account, we can conclude that AONF has a positive effect on the adhesive interaction of CR with glass. We will also attach photographs of the glass samples (Figure 5). We see that the CR sample, like the CR/AONF sample with a concentration of 2% by weight, exhibits an adhesive failure pattern. At the same time, the failure pattern for 0.25% and 1% AONF is adhesive-cohesive, which confirms our hypothesis.

However, when the AONF concentration exceeds 1% by weight, the opposite pattern is observed. The peak of maximum force for glass samples shifts to the left and exhibits a steep decline, indicating a brittle fracture pattern. For steel plates, the peak broadens, with a maximum lower than for the sample with 1% by weight, indicating a brittle fracture pattern. This is indicated by the sharp slope of the curve after the peak.

Figure 4c,d show graphs of the work required to break the adhesive bond between steel plates (c) and glass (d). The graphs reveal a clear optimum concentration (1% AONF by weight).

We also tested strips of the CR composite and CR/AONF in tension. The results are shown in Figure 4e. We see that the sample with the pure CR composite has a gentle slope, indicating the plastic deformation pattern typical of CR composites. With the addition of 0.05% AONF, the slope of the graph steepens, and after the plateau, the graph becomes more linear. This trend in the curve indicates that the CR/AONF composite is stiffer and more brittle than the CR composite. This observation is consistent with the strengthening mechanisms proposed in the previous paragraphs. Interestingly, such a low concentration does not affect the tensile strength, but only changes the deformation pattern. A further increase in the concentration of AONF leads to both a change in the deformation pattern and an increase in tensile strength relative to the reference value. For example, with the addition of 0.25% by weight of AONF, the tensile strength reaches 1.32 MPa, while with 1%, it reaches 1.54 MPa. Considering the slope of the curve in the initial section, it can be concluded that the sample becomes more rigid. The deformation pattern changes at higher concentrations, with the addition of 1% by weight. The AONF curve has a pronounced slope after a steep section, indicating a plastic nature. With the addition of 2% by weight of AONF, the tensile strength decreases to 1.31 MPa, and the curve exhibits a trend characteristic of brittle specimens. The trend of the CR/AONF curve at 1% can be explained by the high degree of load transfer to the AONF blocks and their initial relative sliding. The sliding of AONF in the blocks and the subsequent mobility of the polymer chains influence the deformation, creating a section with a slight slope after the plateau. The influence of AONF also extends to other material properties, such as the yield strength and elastic modulus.

The elastic modulus is defined as the slope of the initial portion of the curve. Figure 4f shows the elastic moduli for the CR composite and the CR/AONF composite. The elastic moduli increase with increasing AONF concentration, consistent with the previously stated theory of increasing stiffness.

The yield strength is a mechanical property of a material that characterizes the stress at which plastic deformations continue to increase without increasing load. It is determined graphically at the intersection of the load–elongation curve with a straight line drawn parallel to the rectilinear initial portion of the stress–elongation curve and intercepting the elongation region corresponding to the relative elongation from the abscissa.

We see that the relative yield strength increases with increasing AONF concentration in the polymer matrix. This is consistent with our earlier assumptions. AONF act as reinforcing components. By redistributing the load, they shift the yield point towards higher forces.

## 5. Thermic Analysis and DMA

AONF can affect the thermophysical properties, thermal stability, and thermal decomposition kinetics of chloroprene rubber. To study the influence of AONF on these parameters and processes, we used TGA, DSC, and DMA methods.

**Figure 6:**
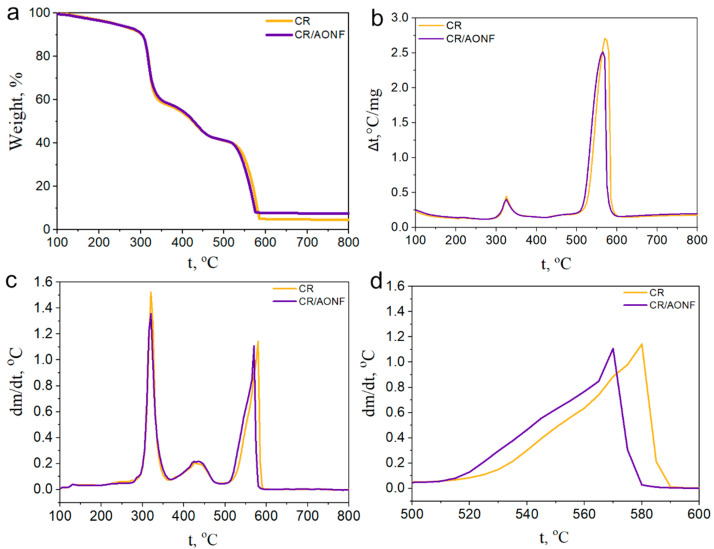
Thermal properties of the CR/AONF composite: (**a**)—TGA; (**b**)—DTGA; (**c**)—DSC; (**d**)—DSC peak.

Figure 6a shows a stepwise loss in mass of both the pure adhesive and the material compounded with aluminum oxide nanofibers. The sequential loss in mass with increasing temperature is associated with several successive processes. The first is the removal of the solvent. The second is the intense stage of CR decomposition: at 300–310 degrees Celsius, the intense stage of CR decomposition begins and a sharp loss in mass is observed (from 90% to 55% on average for all samples). At this stage, autocatalytic pyrolysis of chloroprene chains occurs [39]. The third is polymer carbonization and, finally, complete combustion of the organic residue. This process is typical for CR-based composites and is called high pyrolysis [42]. The presence of aluminum oxide nanofibers results in a higher ash residue than in the pure sample. Otherwise, the curves are identical. Figure 6b illustrates the rate of mass loss (the derivative of mass with respect to temperature) in the materials. Here, one can observe the coincidence of the line of the pure and compounded sample, as well as the peak velocity during carbonization of the material; finally, the combustion peak for the pure sample occurs later than for the sample with AONF. This correlates with the DSC data in Figure 6d, where the combustion reaction of the compounded sample also occurs earlier than that of the pure sample [43]. Together, these data indicate the influence of the high specific surface area of the additive, which accelerates oxidation processes and the removal of reaction products.

In Figure 6c, an exothermic peak near 430 °C is also distinguished. This peak cannot be attributed to the carbonization process—it is caused by the peak near 300–310 °C, and this peak cannot be related to the main oxidation of organic matter, which occurs after 500 °C [44,45]. For many organic compounds, resinification processes are characteristic during intensive decomposition—the formation of oligo- and polyaromatic compounds. In this case, we observe precisely this process. During resinification, the polymer carbonization processes slow down; moreover, ambient oxygen can attach to the forming resins. Indeed, near 400–450 °C in Figure 6a, we see a slowdown in mass loss, and in Figure 6b, an indistinct, blurred bend in the graphs. It is not possible to better isolate this process due to the continuing mass loss during carbonization and the beginning of mass loss from oxidation of the material. Therefore, we can conclude that the exothermic peak near 430 °C is related specifically to vulcanization processes.

Overall, it can be concluded that the addition of aluminum oxide nanofibers influences the key physicochemical processes in the material due to their specific surface area. Aluminum oxide nanofibers accelerate the removal of solvent from the composite, which may be beneficial for a faster bonding process, and they accelerate the oxidation of the organic residue in the material, which is important for reducing energy consumption during recycling of used bonded parts.

**Figure 7:**
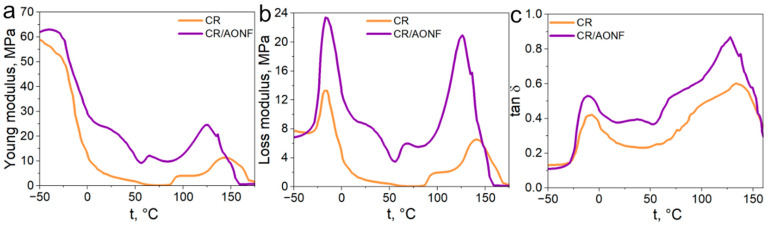
DMA data. Young modulus (**a**), loss modulus (**b**), loss tangent (**c**).

To study the effect of AONF on the phase transitions of CR, we performed DMA in the temperature range from −50 to +160 °C. Figure 7 shows the results of the experiments.

In the glassy state, where the chains are virtually immobile, the presence of AONF acts as a reinforcing component, increasing stiffness. Figure 7a shows elastic moduli of 54 MPa and 62 MPa for the CR and CR/AONF composites.

Furthermore, the addition of AONF increased the glass transition temperature by ~5 °C. This clearly indicates that AONF restricts the mobility of polymer chains. They act as physical crosslinks or entanglements, making the transition from the glassy to the rubbery state more costly and, therefore, requiring a higher temperature.

The broader tan δ (Figure 7c) peak for CR/AONF indicates a more heterogeneous (inhomogeneous) composite structure. Some polymer chains are strongly bound to the filler surface and have limited mobility, which “blurs” the transition.

We also see a reinforcing effect in the highly elastic state. In Figure 7a, we see that the elastic modulus CR is higher than the elastic modulus CR/AONF of the sample throughout the entire test. This effect is explained by the fact that rigid AONF effectively absorb mechanical loads, creating a reinforcing structure within the polymer matrix. This increases the material’s rigidity at operating temperatures.

## 6. Conclusions

In this study, we synthesized high-aspect-ratio AONF and characterized them using SEM, TEM, and XRD. Using the resulting AONFs, we modified a CR composite and subjected it to tensile testing. We also bonded steel plates and glass and tested the adhesive bond in shear.

Shear tests showed that even low AONF concentrations (0.05 wt.%) increased the shear strength by 25% for steel and 35% for glass. The optimal concentration was 1 wt.% AONF, which increased the shear strength by 213% (for steel plates) and 263% (for glass).

We identify several strengthening mechanisms: reinforcement of the CR matrix with AONF, which redistributes the load; the formation of a boundary phase between the CR matrix and AONF with higher fracture energy; and the role of AONF as an obstacle to crack propagation in the CR composite matrix. A further increase in the AONF concentration to 2 wt.% leads to a decrease in shear strength relative to an AONF concentration of 1 wt.%, due to the influence of several simultaneously occurring processes. The first is the displacement of the CR matrix volume, making the CR/AONF composite more brittle; the second is weak adhesion at the interface at high concentrations; and the third is the mutual sliding of AONF within blocks, leading to failure of the entire CR/AONF composite.

Tensile tests of CR/AONF also show an increase in tensile strength with increasing AONF concentration. For example, the tensile strength for the reference CR composite is 0.87 MPa, while for 1% by weight AONF, it is 1.57 MPa, which corresponds to an 80% increase. All mechanical parameters obtained in this study are presented in Table 1.

Optimal selection of the material concentration and method of introduction is critical for the incorporation of AONF. We demonstrated that using a technologically simple method for introducing our synthesized AONF with a high aspect ratio at a concentration of 1% by weight allows for high material strengthening.

This composition retains all the positive properties of the CR matrix while increasing its strength significantly. This composition can be used in applications where the resistance to atmospheric influences, UV, and reagents inherent to CR composites is required, but mechanical properties are insufficient.

The applicability of the modification method is not limited to the CR composite used in this study. The AONF fibers we synthesized can be introduced via pre-dispersion, enabling their use for strengthening adhesives and sealants (with the appropriate solvent selection).

Our results provide an important contribution to the modification of CR-based compositions and demonstrate the potential for AONF applications in this field.

## Figures and Tables

**Table 1:** Technical parameters of CR/AONF composites.

C_AONF_, wt.%	σ, MPa	E, MPa	σ_y_, MPa
CR	0.86	6.4	0.73
0.05	0.95	7.5	0.82
0.25	1.3	10.1	1
1	1.52	17.1	1.1
2	1.25	9.1	1.05

## Data Availability

Data is contained within the article.

## Abbreviations

The following abbreviations are used in this manuscript:

CR	Chloroprene rubber
AONF	Aluminum oxide nanofiber
SEM	Scanning electron microscopy
TEM	Transmission electron microscopy
EDX	Energy-dispersive X-ray spectroscopy
XRD	X-ray diffraction
